# Clinical experiences in the management of critically ill patients with COVID-19 in a designated children’s hospital in China

**DOI:** 10.1007/s12519-023-00718-6

**Published:** 2023-04-15

**Authors:** Gong-Bao Liu, Ying Gu, Ying-Wen Wang, Chuan-Qing Wang, Jian Ma, Mei Zeng, Guo-Ping Lu, Zhong-Lin Wang, Ai-Mei Xia, Jin-Hao Tao, Xiao-Wen Zhai, Wen-Hao Zhou, Hong Xu, Yong-Hao Gui, Guo-Ying Huang, Xiao-Bo Zhang

**Affiliations:** 1 Department of Medical Affairs, Children’s Hospital of Fudan University, Shanghai, China; 2 Department of Nursing, Children’s Hospital of Fudan University, Shanghai, China; 3 Department of Nosocomial Infection Prevention and Control, Children’s Hospital of Fudan University, Shanghai, China; 4 Department of Infectious Disease, Children’s Hospital of Fudan University, Shanghai, China; 5 Department of Critical Medicine, Children’s Hospital of Fudan University, Shanghai, China; 6 Department of Hematology, Children’s Hospital of Fudan University, Shanghai, China; 7 Department of Neonate, Children’s Hospital of Fudan University, Shanghai, China; 8 Department of Nephrology, Children’s Hospital of Fudan University, Shanghai, China; 9 Department of Key Laboratory of Ministry of Health for Neonatal Disease, Children’s Hospital of Fudan University, Shanghai, China; 10 Department of Cardiology, Children’s Hospital of Fudan University, Wanyuan Rd, No. 399, Shanghai, China; 11 Department of Respiratory, Children’s Hospital of Fudan University, Wanyuan Rd, No.399, Shanghai, China

Omicron variant has been the dominant epidemic variant of novel coronavirus in the world since 2021. Studies have shown that the incidence of severe or critical cases with coronavirus disease 2019 (COVID-19) is much lower than that of the previous novel coronavirus variants [1, 2]; however, it is still a major challenge to treat critical pediatric patients with COVID-19, especially for those with underlying diseases. There are different points between the WHO and National Health Commission of China (NHCC) in the definition of severe or critical disease with COVID-19 [3, 4] (Supplement Table 1). In China, all designated hospitals refer to the guidelines for the treatment of COVID-19 issued by the NHCC [4].

Underlying diseases are common in critical pediatric patients with COVID-19 [5]. Twenty-one underlying diseases have been reported as high-risk factors for critical illness, such as asthma, cancer, cerebrovascular diseases, chronic kidney diseases, chronic lung diseases, and diabetes mellitus [6]. Three critical patients with underlying diseases (malignant tumor, acute brain failure, Rett syndrome) were treated in our hospital from April to May 2022 (Supplement Table 2). Herein, we want to share the experiences of treating critical pediatric patients with COVID-19.

## Assessment and monitoring

**Fig. 1:**
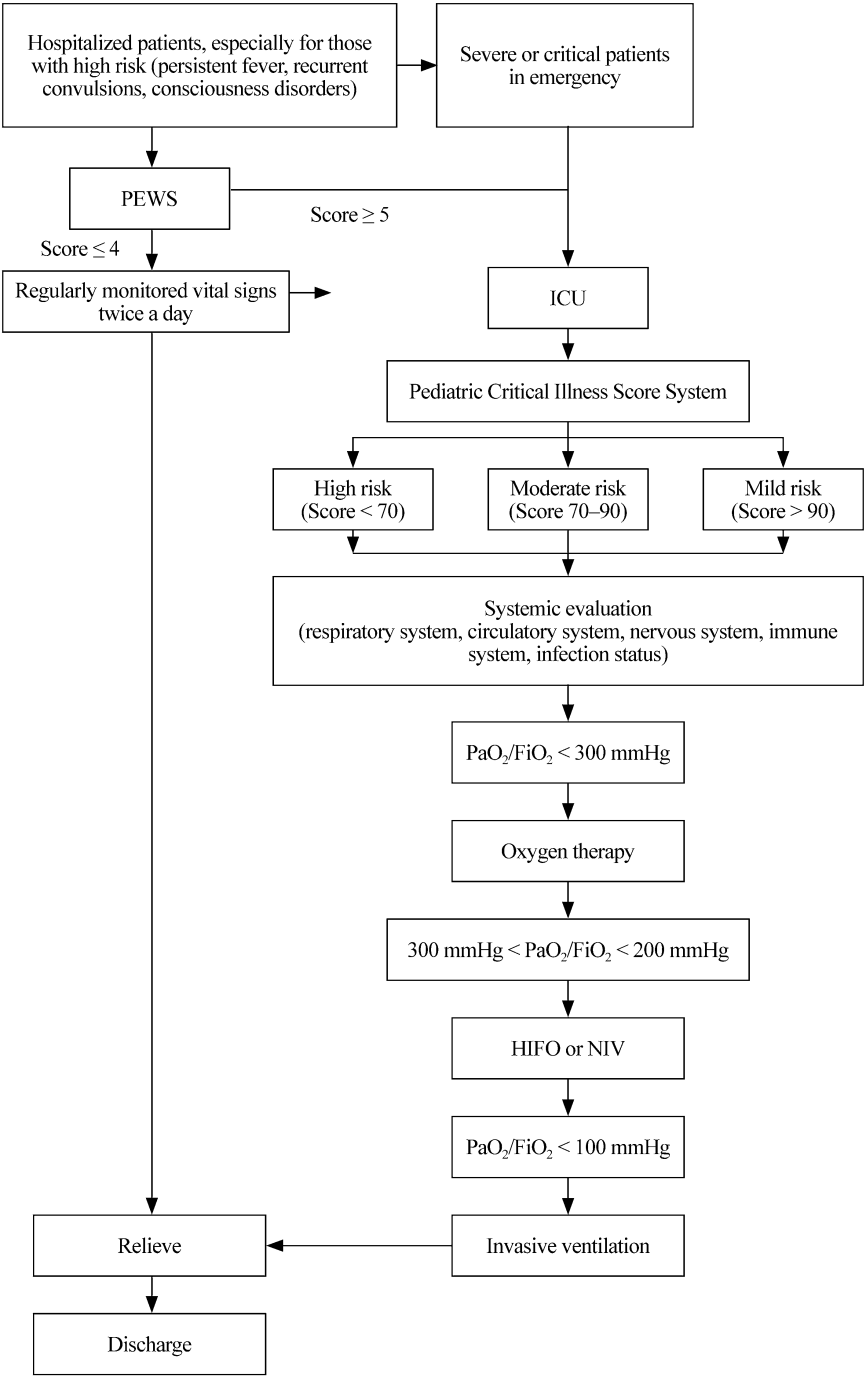
Flowchart of inpatients. *PEWS* Pediatric Early Warning Score, *ICU* intensive care unit, *HIFO* nasal high flow oxygen, *NIV* noninvasive ventilation

In addition to some routine assessments, such as fall risk, pressure injury and nutritional status, each patient hospitalized with COVID-19 was regularly monitored for vital signs, including pulse oximetry, twice a day. Anyone who meets the criteria of severe or critical illness [4] will be transferred to the ICU immediately. The first score of the Pediatric Early Warning Score (PEWS) system [7] is performed at admission and then every 4 hours until 24 hours after admission. Patients with a score ≥ 5 will be transferred to the ICU. Critical patients were evaluated by the Pediatric Critical Illness Score (PCIS) system [8] at admission to the ICU. A score of > 90 indicates a moderate severity disease, while a score of < 70 means an extremely critical disease. Furthermore, the assessment by the PCIS is repeated when the illness deteriorates to the most serious condition or when the patient is relieved and transferred out of the ICU (Fig. 1). Each patient in the ICU should be systemically evaluated, including the condition of underlying diseases. Special attention should be given to patients with persistent fever for longer than 3 days, recurrent convulsions and consciousness disorders.

## Treatment of critical disease

Many studies have reported that the prone position is useful for improving respiratory dysfunction in adults with severe COVID-19 [9, 10]. However, there are fewer reports on the application of prone position ventilation in critical children with COVID-19 [11]. Prone position is recommended for medical institutions with related experiences [12]. Prone position for 12–16 hours in critically ill children with careful nursing is useful according to our practical experience.

Patients have differentiated respiratory support according to the severity of the condition [3]. In any case, patients with PaO_2_/FiO_2_ < 300 mmHg should receive oxygen therapy immediately. Nasal high flow oxygen (HIFO) or non-invasive ventilation (NIV) is suitable for PaO_2_/FiO_2_ < 200 mmHg. Those with PaO_2_/FiO_2_ < 150 mmHg should receive invasive ventilation as soon as possible [4]. However, PaO_2_/FiO_2_ is not the only reference index to measure the degree of respiratory dysfunction in patients, and it is necessary to make a comprehensive assessment based on the clinical manifestation. Patients with HIFO or NIV should be carefully monitored; in cases whose symptoms of respiratory distress deteriorate or do not improve after a short trial (about one hour), endotracheal intubation followed by invasive ventilation should not be delayed. SpO_2_ ≥ 94% is reported as the target of oxygen therapy during resuscitation [13]. The critically ill patient with Rett syndrome in our hospital underwent HIFO for seven days but PaO_2_/FiO_2_ was still be as low of 93.8 mmHg. Therefore, invasive ventilation was used due to the deterioration of acute respiratory distress syndrome (ARDS). For critically ill patients with underlying diseases of neuromuscular distress, the function of respiratory muscles is weakened, and airway clearance techniques are recommended under restricted conditions [14]. Both high-frequency chest wall oscillation (HFCWO) and mechanical insufflator–exsufflator (MIE) have been proven to improve oxygenation in adults with COVID-19 [15, 16]. However, the experiences in using HFCWMO and MIE in critically ill children are limited. According to our own practical experiences, bronchoscopic airway flushing with the assistance of HFCWO and MIE is useful in critically ill patients with neuromuscular dysfunction. After seven days of this joint treatment, the patient with Rett syndrome successfully weaned from mechanical ventilation. Severe COVID-19-related ARDS, myocarditis, or multisystem inflammatory syndrome in children are all indications of extracorporeal membrane oxygenation (ECMO) [17]. Overall, the basic principles of ECMO for children with COVID-19 are similar to those for children with other diseases.

Paxlovid was emergently authorized by the Chinese Food and Drug Administration for the treatment of COVID-19 in February 2022 in China. It is recommended for patients with non-severe COVID-19 at the highest risk of hospitalization, while the indication was restricted to adults and some children (≥ 12 years, weighing ≥ 40 kg) [18]. The use of paxlovid in younger children without oxygen therapy has also been reported [19, 20]. However, the viral shedding times were not significantly reduced in age-matched controls [19]. We had a trial of paxlovid in three critically ill children with different underlying diseases, but it did not reduce the viral shedding time as expected. The longest viral shedding time was 21 days. Dexamethasone or alternative steroid regimens (prednisolone, methylprednisolone) are recommended for severe or critically ill children [21]. However, the use of steroid regimens should be more cautious for those with malignant tumors.

The treatment of critically ill children with septic shock focuses on early recognition, rapid fluid resuscitation, and the use of antibiotics and vasopressors [3]. In the process of fluid resuscitation, the circulatory system should be closely observed to avoid excessive volume load to aggravate ARDS. Continuous renal replacement therapy (CRRT) should be started when severe circulatory volume overload occurs with failure of diuretic treatment. We applied ECMO with CRRT to a critically ill child with malignant tumor, ARDS and septic shock, but this advanced life support only lasted for five days due to the end-stage of the tumor.

## Additional management

Common critical care equipment, such as defibrillators, ventilators, high-flow oxygen instruments, bedside ultrasound, bedside electrocardiogram, bronchoscopes, etc., should be prepared in isolation ICUs in advance. At least one ECMO and one CRRT instrument are both available at any time; and further allocation plan of the hospital should be fully prepared according to the number of critically ill patients. Pediatric Advanced Life Support training certification is the other essential requirement of critical care teams. The role of respiratory therapists in patients' oxygen therapy has been widely recognized. The collaboration of this multidisciplinary team is crucial. Previous therapy of underlying diseases should be continued or modified by multidisciplinary collaboration according to the patient’s severity of condition.

In conclusion, due to the various underlying diseases of children, each critical patient needs close cooperation of the whole multidisciplinary team. Identifying the high-risk factors for critical illness, continuous monitoring, early recognition of disease deterioration and timely intervention, and scientific allocation of various equipment and human resources are all important parts of successful management.

## Supplementary Information

Below is the link to the electronic supplementary material.

Supplementary file1 (DOCX 27 KB)

## Data Availability

All data generated or analyzed during this study are included in this published article (and its supplementary information files).
