# Supplementary material for: Clinical experiences in the management of critically ill patients with COVID-19 in a designated children’s hospital in China
Source: World J Pediatr. 2023 Apr 15;19(6):605–8. doi: 10.1007/s12519-023-00718-6 (PMC10105610; doi:10.1007/s12519-023-00718-6)
Supplement: Supplementary file 1 — Supplementary file1 (DOCX 27 KB) [file 12519_2023_718_MOESM1_ESM.docx]

**Supplement Table1. Comparison of two classification between WHO and NHCC**

| Classification | WHO | NHCC* ( the Ninth Edition ) |
| --- | --- | --- |
| Mild disease | Patients without pneumonia or hypoxia | Patients without pneumonia or hypoxia |
| Moderate disease | Patients with pneumonia.  No signs of severe pneumonia.  Fast breathing. | Patients with pneumonia |
| Severe disease | Patients with pneumonia and at least one of following symptom:   1. SpO_2_<90% 2. Symptom of severe respiratory distress or other danger sign (severe chest wall indrawing, inability to breastfeed of drink, lethargy, unconsciousness, or convulsions ) | Conform to any of the following:   1. Fever lasts for more than three days. 2. Fast breathing 3. SpO_2_≤93% 4. Symptom of severe respiratory distress or other danger sign(inability to breastfeed or drink with sign of dehydration, nasal ale flap, chest wall indrawing, unconsciousness, or convulsion) |
| Critical disease | 1. ARDS^*^ 2. Sepsis 3. Septic shock 4. Acute thrombosis 5. MIS-C^*^ | 1. ARDS with mechanical ventilation 2. Shock 3. MODS^*^ with life sustaining treatment in ICU^*^ |

**NHCC*, National Health Commission of China,

*ARDS* ,Acute respiratory distress syndrome, *MIS-C ,*Multi-system inflammatory syndrome

*MODS:* Multiple organ dysfunction syndrome *ICU*, Intensive care unit

**Supplement Table2. History of three critical patients treated in our hospital**

|  | Age | Underlying disease | PCIS at admission of ICU | Respiratory support | Other advanced life support | Airway clearance technique | Prone position | Paxlovid |
| --- | --- | --- | --- | --- | --- | --- | --- | --- |
| Case 1 | 7y | Rett syndrome | 90 | HIFO→IV | None | √ | √ | √ |
| Case 2 | 3y | Acute brain failure | 68 | IV | None | None | None | √ |
| Case 3 | 11y | Malignant tumor with multiple metastases | 80 | IV | CRRT+ECMO | None | √ | √ |

*PCIS* Pediatric Critical Illness Score, *HIFO* High flow oxygen, *IV* Invasive ventilation,

*CRRT* Continuous renal replacement therapy, *ECMO* Extracorporeal membrane oxygenation
